# Proper Immune Response Depends on Early Exposure to Gut Microbiota in Broiler Chicks

**DOI:** 10.3389/fphys.2021.758183

**Published:** 2021-10-15

**Authors:** Denise R. Rodrigues, Kim M. Wilson, Lisa R. Bielke

**Affiliations:** ^1^Department of Animal Sciences, The Ohio State University, Columbus, OH, United States; ^2^Department of Inspection of Animal Products, Ministry of Agriculture, Livestock and Food Supply (MAPA), Brasília, Brazil

**Keywords:** immune programming, probiotics, *Enterobacteriaceae*, lactic acid bacteria, intestinal microbiome, innate immune system, proteome, pathways

## Abstract

The successional changes in the early intestinal microbiota occur concomitantly with the development, expansion, and education of the mucosal immune system. Although great attention of researchers has been focused on understanding the linkage between microbiota and immune functions, many essential details of the symbiotic relationship between the intestinal pioneer microbiota and the avian immune system remain to be discovered. This study was conducted to understand the impact of different early life intestinal colonizers on innate and adaptive immune processes in chicks and further identify immune-associated proteins expressed in the intestinal tissue. To accomplish it, we performed an *in ovo* application of two apathogenic *Enterobacteriaceae* isolates and lactic acid bacteria (L) to determine their influences on the intestinal proteome profile of broilers at the day of hatch (DOH) and at 10 days old. The results indicated that there were predicted biological functions of L-treated chicks associated with the activation and balanced function of the innate and adaptive immune systems. At the same time, the *Enterobacteriaceae-*exposed birds presented dysregulated immunological mechanisms or downregulated processes related to immune development. Those findings suggested that a proper immune function was dependent on specific gut microbiota exposure, in which the prenatal probiotic application may have favored the fitting programming of immune functions in chicks.

## Introduction

In recent years, attention has focused on understanding the linkage between microbiota and immune functions. Such an intimate relationship creates mechanisms for mutual benefits to both microbes and the host (Chow et al., 2010). At homeostasis, this mutualistic partnership enables the maintenance of microbial tolerance in the intestinal ecosystem, and, in turn, the proper microbiota colonization contributes to the development, maturation, and function of the immune system (Bar-Shira et al., 2003; Kelly et al., 2007; Brisbin et al., 2008; Chung et al., 2012).

Nevertheless, the cooperative arrangements between gut microbiota and host mucosal immunity are constantly threatened during the early life of chickens. Evidence has suggested that undesirable microbial colonization during the prenatal period may negatively influence the function and expansion of the immune system in broilers (Bar-Shira et al., 2003; Rodrigues et al., 2020a). Previous work has also demonstrated that perturbation of the intestinal pioneer microbiota with antibiotic exposure affects early immune programming and has been shown to boost negative antibody response in hens after cessation of antibiotic treatment (Simon et al., 2016). Besides, our previous reports indicated that pre-hatch colonization by *Enterobacteriaceae* promoted immune dysregulation and chronic inflammation (Rodrigues et al., 2020a,b; Wilson et al., 2020). Another example of dysregulated immune response triggered by early disturbance of enteric microbiota in chicks was shown by Schokker et al. (2010). It was revealed that early colonization by *Salmonella enterica* serotype Enteritidis delayed the morphological processes of the jejunum, thereby interrupting the spatial-temporal development of the immune system in chicks. On account of this fact, intestinal dysbiosis during the early post-hatch phase in chicks may have short- and long-term consequences on immune responses. Against this background, many essential details of the symbiotic relationship between the intestinal pioneer microbiota and the avian immune system remain to be discovered, and an important outstanding question was whether a proper early immune response depends on host-specific gut microbiota.

Recently, the emergence of *in ovo* techniques made it possible to manipulate the intestinal bacteria colonization before chicks have even been hatched or exposed to farm environments (Pedroso et al., 2016; Roto et al., 2016; Teague et al., 2017). Our lab has previously used the *in ovo* technique as an experimental model to address how early intestinal colonization shapes the microbiome composition in hatching chicks (Wilson et al., 2020). The technique of delivering various nutrients, supplements, immunostimulants, vaccines, and drugs *via* the *in ovo* route is gaining wide attention among researchers for boosting the production performance and immunity and for safeguarding the health of poultry (Saeed et al., 2019; Alagawany et al., 2021; Hassan et al., 2021).

In order to better understand the impact of different early life intestinal colonizers on innate and adaptive immune processes in chicks and further identify immune-associated proteins expressed in the intestinal tissue, we performed an *in ovo* application of two apathogenic *Enterobacteriaceae* isolates and lactic acid bacteria to determine their influences on the intestinal proteome profile of broilers at the day of hatch (DOH) and 10 days of age. Proteomics pathway enrichment and Gene Ontology (GO) function annotation analyses were performed to reveal the effect of different pioneer intestinal colonizers on innate and adaptive immune processes in chicks.

## Materials and Methods

### Study Design

The trial was performed on Ross 708 fertile eggs obtained from a local hatchery and incubated under standard conditions at the poultry research farm of the Ohio Agricultural Research and Development Center. All eggs were in the same incubator prior to inoculation. Once eggs were confirmed fertile, at embryonic day 18, inoculations containing one of the following: 0.9% sterile saline (S), ~10^2^ cells of *Citrobacter* (CF), *Citrobacter* 2 (C2), or a lactic acid bacteria probiotic (L) were administered *via in ovo* injection into the amnion. After inoculation, the eggs were allocated by treatments into separate benchtop hatchers (Hova-Bator model 1602N, Savannah, GA, USA). Each inoculation treatment was separated into three hatchers, which contained up to 30 eggs. The chicks hatched between 48 and 72 h post-inoculation. The hatchability ranged from 86 to 100% among the *in ovo* treatments (Wilson et al., 2020). The bacterial inoculum was selected from our previous trial (Bielke et al., 2003), and the homology of strains was confirmed by next-generation sequencing. The L isolate was composed of a mixed culture of *Lactobacillus salivarius* and *Pediococcus* ssp. The CF strain was composed of *Citrobacter freundii*, and the C2 was identified as *Citrobacter* spp. Bacterial inoculum was prepared as described by Wilson et al. (2020). All experimental procedures were approved by the Institutional Animal Care and Use Committee (IACUC) of the Ohio State University.

### Sample Collection

Once all chicks were hatched, 10 chicks were randomly chosen from among the hatchers within each of the treatments (*n* = 40) and were immediately euthanized *via* cervical dislocation. The intestine was aseptically removed, from the duodenum to the cloaca. Tissues were placed in individual 2 ml tubes and flash-frozen in liquid nitrogen. Immediately post-hatch, the remaining 128 chicks were placed into treatment-separate brooder battery cages and had *ad libitum* access to a standard corn-soy diet and water (Nutrient Requirements of Nutrient Requirements of Poultry., 1994). At 10 days post-hatch, 12 chicks per treatment were randomly selected for ileal proteome analysis. The tissue of the region above the ileocecal junction, designated as lower ileum, was aseptically collected (*n* = 45). Since there were three mortalities in the CF, only nine birds were sampled for this treatment. Ileum tissue was also frozen and stored at −80°C until further use.

Once intestinal samples were thawed, a cumulative total of 0.1 g was individually placed into 5 ml of buffer (8 M urea/2 M thiourea, 2 mM DTT, 50 mM Tris, 5% SDS, pH 6.8). The extraction protocol is a modified version described previously by Iqbal et al. (2004) and Kong et al. (2016). In brief, samples were homogenized for 5 s (PRO250 Homogenizer; Pro Scientific, Oxford, CT, USA), and then, 500 μl of the solution was placed in 2 ml tubes containing 0.1 g stainless steel beads (SSB14B; Next Advance, Averill Park, NY, USA). Samples were homogenized for 3 min total in 30-s intervals (MiniBeadbeater-16, Model 607; BioSpec Products, Bartlesville, OK, USA) and centrifuged at 4°C at 14,000 *g* for 20 min. The supernatant was collected, aliquoted, and placed at −80°C until further use.

To ensure proper extraction, the concentration of total protein was quantified with the Bradford assay (Bradford reagent; VWR, Suwanee, GA, USA) and a standard bovine serum albumin curve (VWR, Suwanee, GA, USA) on a Synergy HTX multi-mode plate reader (BioTek Instruments, Winooski, VT, USA). Samples were mixed to create pooled samples from two birds per treatment/time and were sent to the Ohio State University Proteomics Core lab for performing in solution digestion and mass spectrometry *via* established methods.

### Proteomics Analyses

Samples were precipitated with 25% (w/v) trichloroacetic acid (TCA) and then resuspended in 50 mM ammonium bicarbonate. A total of 5 ml of DTT (5 μg/μl in 50 mM ammonium bicarbonate) was added, and the samples were incubated at 56°C for 15 min. After incubation, 5 μl of iodoacetamide (15 mg/ml in 50 mM ammonium bicarbonate) was added, and the samples were kept in the dark at room temperature for 30 min. Sequencing grade-modified trypsin (Promega, Madison, WI, USA) prepared in 50 mM ammonium bicarbonate was added to each sample with an estimation of 1:20/1:100 enzyme-substrate ratio set at 37°C overnight. The reaction was quenched the following day by adding acetic acid for acidification. Once samples were quenched, the peptide concentration was measured by Nanodrop (Thermo Scientific Nanodrop 2000; Thermo Scientific, Waltham, MA, USA).

Capillary-liquid chromatography-nanospray tandem mass spectrometry (Capillary-LC/MS/MS) of global protein identification was performed on a Thermo Fisher Fusion mass spectrometer (Thermo Scientific, Waltham, MA, USA). Samples were separated on a Thermo Nano C18 column (UltiMate™ 3000 HPLC system; Thermo Scientific, Waltham, MA, USA). The MS/MS data sequences were scanned, and the scan sequence was based on the preview mode data-dependent TopSpeed™ method with CID and ETD as fragmentation methods. The raw data were searched on Sequest *via* Proteome Discoverer (Proteome Discoverer™ software; Thermo Scientific, Waltham, MA, USA). The data were searched against the most recent Uniprot *Gallus gallus* database for the identification of proteins. Only proteins with < 0.05 false discovery rate (FDR) were reported. Proteins with a Mascot score of 50 or higher with a minimum of two unique peptides from one protein having a -b or -y ion sequence tag of five residues or better were accepted. Any modifications or low score peptide/protein identifications were manually checked for validation.

### BioInformatics and Statistical Analysis

Label-free quantitation was performed using the spectral count approach, in which the relative protein quantitation is measured by comparing the number of MS/MS spectra identified from the same protein in each of the multiple LC/MSMS datasets. Comparisons between *in ovo* bacterial treatments and S control group were performed in Scaffold (Scaffold 4.8.4; Proteome Software, Portland, OR, USA). The student's *t*-test (*P* < 0.05) was performed to identify significance across the fold-change values. Differentially expressed proteins (DEPs) were assigned at level *P* ≤ 0.1.

From the significant proteins were performed GO annotation enrichment terms analysis using the STRING v10.0 database (https://string-db.org). Pathway enrichment was assessed using upregulated DEPs based on Reactome databases. Then to evaluate the role of overexpressed proteins in the immune system, we searched for immune pathways based on biological processes using Reactome pathways (https://reactome.org/). *P* < 0.05 was set as the threshold value, and FDR was corrected using the Benjamini-Hochberg method. Immune-related DEPs were identified and their predicted functional partners were searched (STRING v10.0 database). Then, functional clusters in the protein-protein interaction (PPI) networks were determined. Active interaction sources, including experiments, databases, co-occurrence, and co-expression, as well as species limited to “*Gallus gallus*,” and an interaction score > 0.4 were applied to construct the PPI networks.

## Results

### Identification of DEPs

A total of 78, 107, and 39 proteins were identified as DEPs in the ileum of L, CF, and C2 treatments, based on the set threshold (*P* ≤ 0.1) at DOH. By 10 days of age, a total of 61, 44, and 63 proteins showed differential expression in L, CF, and C2, respectively, compared with the *in ovo* S control.

### Functional Enrichment Analysis

**Figure 1:**
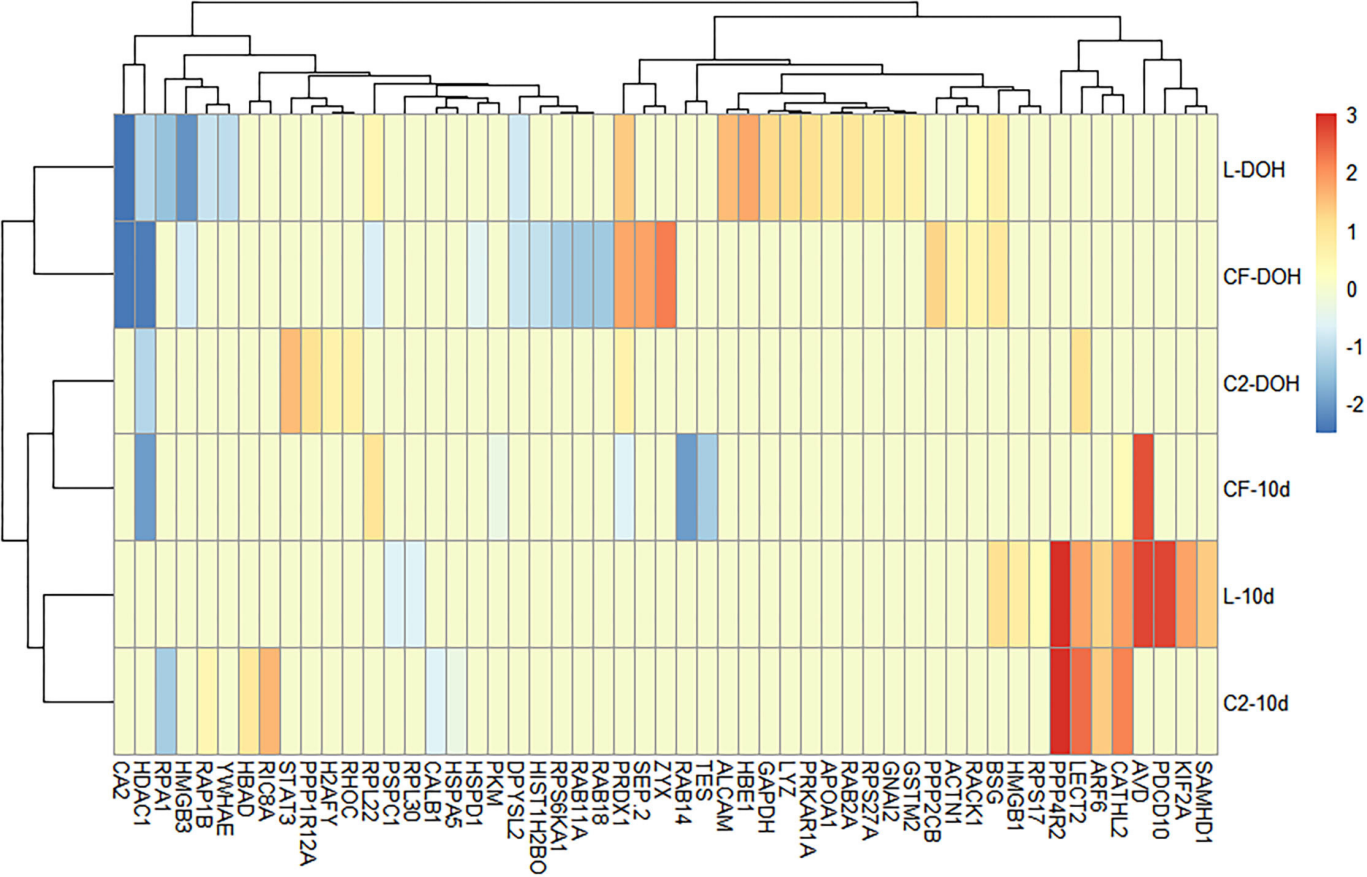
Differentially expressed proteins (DEPs) related to immune processes based on Gene Ontology analysis. Heatmap plot represents the log2-fold changes of proteins expressed intestine samples of broilers treated with lactic acid bacteria (L), *Citrobacter freundii* (CF) or *Citrobacter* spp. (C2) in relation to control treatment at the day of the hatch (DOH) and 10 days of age (10d). Blue shades represent downregulation, while red shades indicate upregulation of the particular protein. Light yellow cells indicate no expression.

To investigate whether different early colonizers in the gastrointestinal tract (GIT) could affect biological processes linked to the immune system, GO enrichment analyses were performed for overexpressed and underexpressed proteins in each dataset. The top 20 enriched GO terms, relative to the control, were exhibited for each *in ovo* treatment at two time points. Proteins associated with response to stimulus and immune annotation roles were displayed in Figure 1.

**Figure 2:**
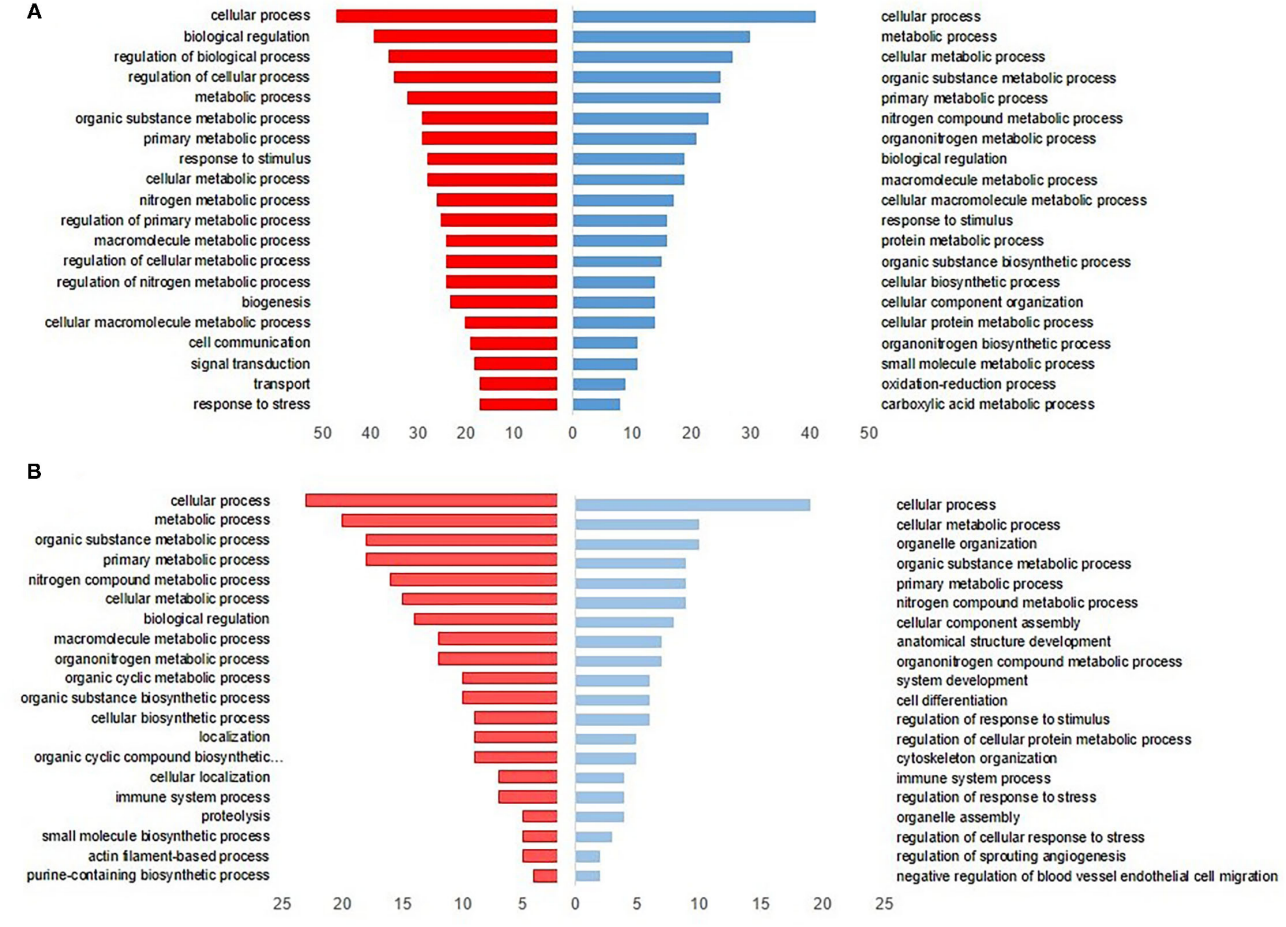
Gene Ontology (GO) enrichment analysis of intestinal differentially expressed proteins from broilers exposed to lactic acid bacteria (L) *in ovo* **(A)** at the day of hatch and **(B)** by 10 days of age. The blue bars to the right indicate the top 20 GO-enriched terms by upregulated proteins. The red bars to the left represent the top 20 GO terms enriched by downregulated proteins, while the axis at the bottom is the number of proteins in each biological process.

The upregulated DEPs in L revealed that the major biological processes at DOH were related to protein and cellular metabolic process, cellular organization, and response to a stimulus (Blue bars in Figure 2A). On the 10th day, cellular differentiation and assembly, regulation of stress response, and immune system process were enriched. In terms of downregulated DEPs in the L dataset, the most enriched processes were biological regulation, cell communication, and response to stress at DOH. While by 10 days of age, proteolysis, cellular metabolic process, and immune system process were overrepresented (Red bars in Figure 2B).

**Figure 3:**
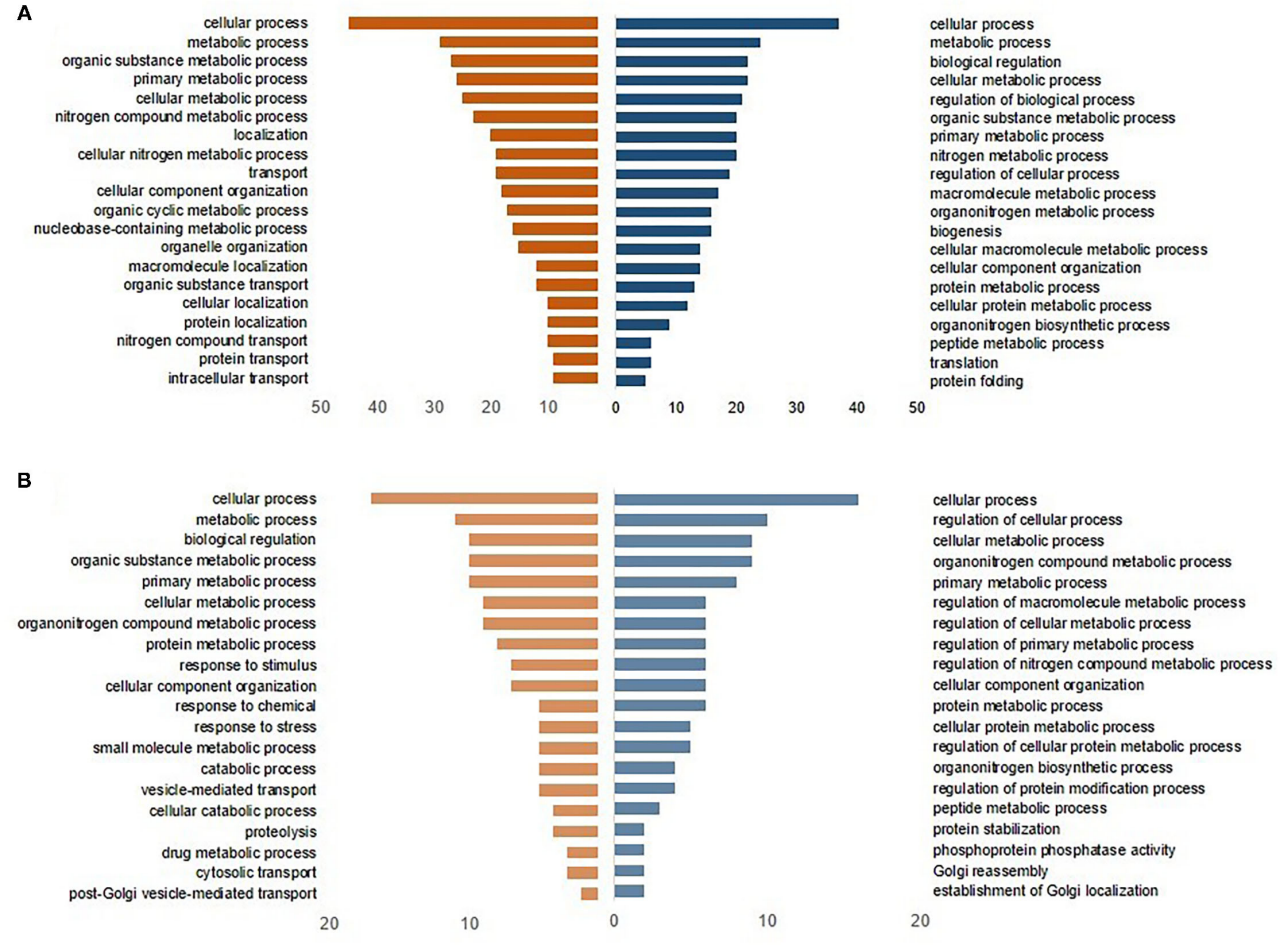
Gene Ontology (GO)-enriched terms of intestinal differentially expressed proteins from broilers exposed to *Citrobacter freundii* (CF) *in ovo* **(A)** at the day of hatch and **(B)** by 10 days of age. The blue bars to the right indicate the most GO-enriched terms by upregulated proteins. The orange bars to the left represent GO terms enriched by downregulated proteins, while the axis at the bottom is the number of proteins in each biological process.

The upregulated DEGs in CF were involved in different GO terms, including cellular and metabolic processes, and protein folding at DOH (Figure 3A), whereas terms associated with protein stabilization and regulation of cellular were evident by 10 days of age (Figure 3B). Conversely, the downregulated DEPs in CF were related to transport, localization, and cellular organization component at DOH (Figure 3A). By 10 days of age, the GO terms were allied to the catabolic process and response to a stimulus (Figure 3B).

**Figure 4:**
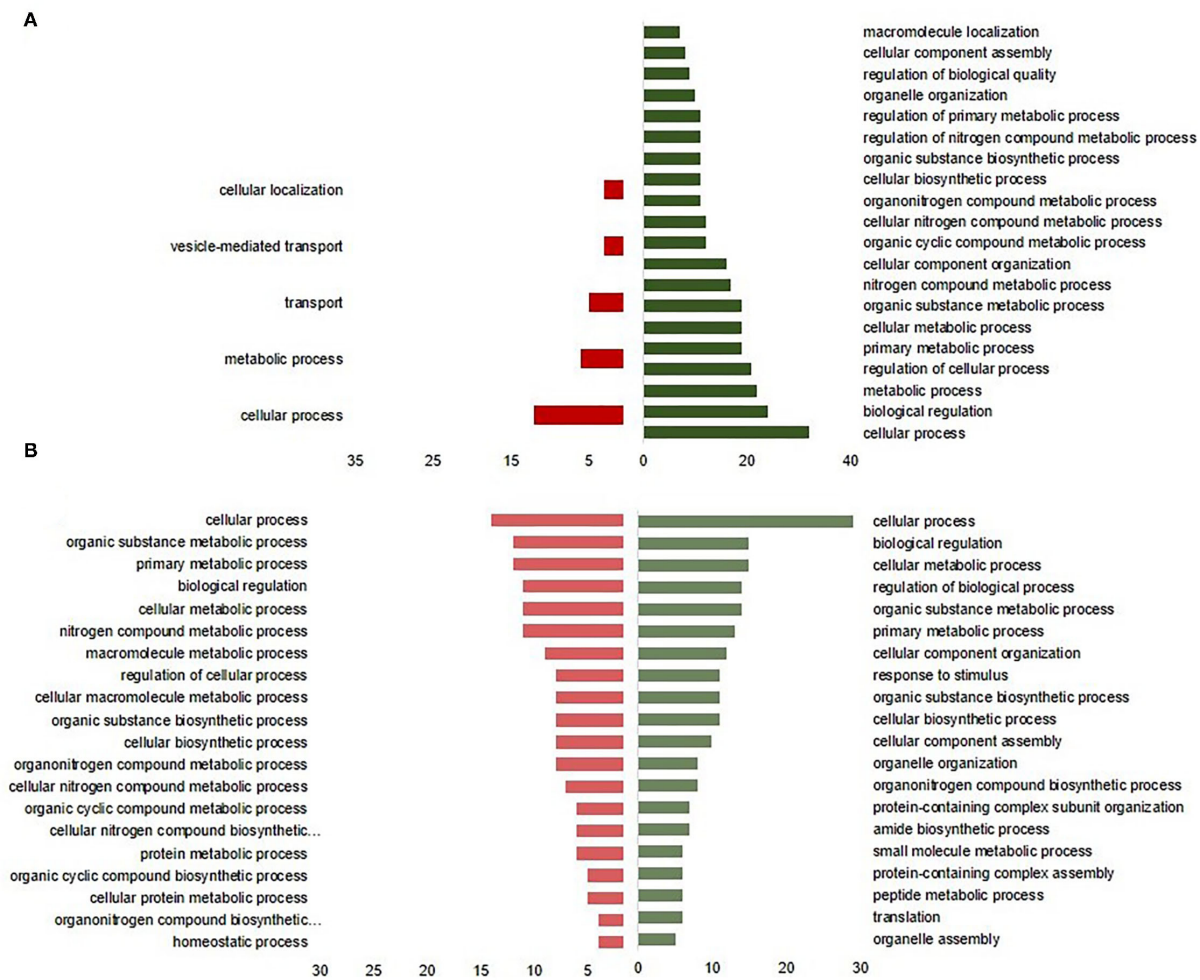
Enrichment of biological process of intestinal differentially expressed proteins from broilers exposed to *Citrobacter* spp. (C2) *in ovo* **(A)** at the day of hatch and **(B)** by 10 days of age. The green bars to the right indicate the most GO-enriched terms by upregulated proteins. The red bars to the right represent GO terms enriched by downregulated proteins, while the axis at the bottom is the number of proteins attributed to each GO term.

The most significant GO annotation based on the overexpressed proteins in C2 was associated with a cellular process, biological regulation, and organelle organization at DOH (Figure 4A), whereas it was associated with cellular metabolic process, primary metabolic process, and response to a stimulus at 10 days of age (Figure 4B). The underrepresented proteins enhanced only a few terms at DOH, including cellular process and transport. Biological functions enriched by 10 days of age were related to protein metabolic, homeostatic, and cellular protein metabolic processes (Figure 4B).

### Pathways With DEPs

The five most significant pathways in L at DOH included amyloid fiber formation, glutathione conjugation, cellular responses to stress, HSF1 activation, and cellular responses to external stimuli (Table 1). The enriched pathways by 10 days of age were muscle contraction, smooth muscle contraction, kinesins, axon guidance, and COPI-dependent Golgi-to-ER retrograde traffic.

**Table 1:** The five most significant pathways based on the upregulated proteins expressed in intestinal samples of broilers treated with lactic acid bacteria (L), *Citrobacter freundii* (CF) or *Citrobacter* spp. (C2) in relation to control treatment at the day of the hatch (DOH) and 10 days of age (10d).

**Pathway name**	**DOH**			**10d**		
	**Ratio**	***P***	**FDR**	**Ratio**	***P***	**FDR**
**L treatment**						
Amyloid fiber formation	0.006	4.54e−06	0.003	–	–	–
Glutathione conjugation	0.005	1.93e−05	0.007	–	–	–
Cellular responses to stress	0.036	3.27e−05	0.008	–	–	–
HSF1 activation	0.003	5.94e−05	0.011	–	–	–
Cellular responses to external stimuli	0.044	1.75e−04	0.025	–	–	–
Muscle contraction	–	–	–	0.018	1.60e−04	0.022
Smooth muscle contraction	–	–	–	0.04	1.99e−04	0.022
Kinesins	–	–	–	0.05	3.69e−04	0.027
Axon guidance	–	–	–	0.041	0.001	0.056
OPI-dependent Golgi-to-ER retrograde traffic	–	–	–	0.008	0.001	0.06
**CF treatment**						
Syndecan interactions	0.002	1.08e−05	0.006	–	–	–
Integrin cell surface interactions	0.006	5.05e−05	0.013	–	–	–
Assembly and cell surface presentation of NMDA receptors	0.003	7.59e−05	0.013	–	–	–
Cellular responses to external stimuli	0.044	1.15e−04	0.014	–	–	–
Extracellular matrix organization	0.023	1.32e−04	0.016	–	–	–
P75NTR negatively regulates the cycle via SC1	–	–	–	4.24e−04	5.80e−05	0.009
Phase II conjugation of compounds	–	–	–	0.018	9.61e−05	0.009
Glutathione conjugation	–	–	–	0.005	2.54e−04	0.016
Acetylation	–	–	–	0.001	4.08e−04	0.019
FOXO-mediated transcription	–	–	–	0.008	0.001	0.038
**C2 treatment**						
Toll-like receptors (TLRs) by endogenous ligand	0.002	1.54e−5	4.17e−3	–	–	–
Signaling by RAF1 mutants	0.003	5.09e−5	4.17e−3	–	–	–
Paradoxical activation of RAF signaling by Kinase inactive BRAF	0.004	7.39e−5	4.17e−3	–	–	–
Signaling by moderate kinase activity BRAF mutants	0.004	7.39e−5	4.17e−3	–	–	–
Signaling downstream of RAS mutants	0.004	7.39e−5	4.17e−3	–	–	–
Cellular response to starvation	–	–	–	0.012	1.58e−6	2.77e−4
Response of EIF2AK4 (GCN2) to amino acid deficiency	–	–	–	0.008	0.000002	2.77e−4
Eukaryotic translation elongation	–	–	–	0.007	2.05e−5	1.61e−3
GRB2:SOS provides linkage to MAPK signaling for Integrins	–	–	–	0.001	4.05e−5	1.61e−3
L13a-mediated translational silencing of Ceruloplasmin expression	–	–	–	0.008	4.43e−5	1.61e−3

*FDR, false discovery rate*.

The most relevant predicted pathways in CF were syndecan interactions, integrin cell surface interactions, assembly and cell surface presentation of NMDA receptors, cellular responses to external stimuli, and extracellular matrix organization at DOH (Table 1). By 10 days of age, pathways associated with P75NTR negatively regulate the cycle *via* SC1, and phase II conjugation of compounds, glutathione conjugation, acetylation, and FOXO-mediated transcription were enriched.

The inoculation of C2 affected pathways related to the regulation of Toll-like receptors (TLRs) by endogenous ligand, signaling by RAF1 mutants, paradoxical activation of RAF signaling by kinase inactive BRAF, signaling by moderate kinase activity BRAF mutants, and signaling downstream of RAS mutants at DOH; whereas cellular response to starvation, response of EIF2AK4 (GCN2) to amino acid deficiency, eukaryotic translation elongation, GRB2:SOS linkage to MAPK signaling for integrins, and L13a-mediated translational silencing of ceruloplasmin expression was activated at 10 days of age (Table 1).

**Figure 5:**
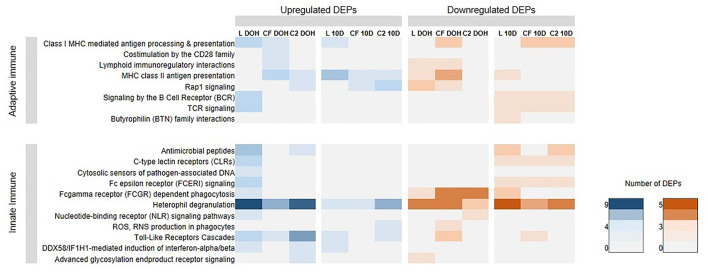
Reactome Pathways analyses, at the day of hatch (DOH) and 10 days of age (10d), related to immune response based on differentially expressed proteins (DEPs) in intestinal samples of broilers treated with lactic acid bacteria (L), *Citrobacter freundii* (CF) or *Citrobacte*r spp. (C2).

The presence of several immune genes prompted us to investigate whether the early exposure to different bacterial isolates could affect the immune biological processes in the GIT of broilers. Then, we performed the broad immune response pathways in Reactome for up- and downregulated proteins. Analyses showed substantially more upregulated DEPs in L treatment that accomplishes functions on immune signaling than the other *in ovo* treatment by DOH (Figure 5). Accordingly, an enrichment of heterophil degranulation (*P* = 0.0005; FDR = 0.034) and antimicrobial peptides pathways (*P* = 0.022; FDR = 0.129) at DOH was observed (Figure 5). By 10 days of age, MHC class II antigen presentation was overexpressed in L treatment (*P* = 0.003; FDR = 0.076). Using downregulated DEPs, Reactome analyses showed that Rap1 signaling was enriched in L at DOH (*P* = 0.004; FDR = 0.053; Figure 5), while the pathways heterophil degranulation (*P* = 0.018; FDR= 0.223) and Butyrophilin (BTN) family interactions (*P* = 0.037; FDR= 0.223) were significant at 10 days of age.

While there was not any pathway enhanced with upregulated DEPs at DOH in CF, the pathway Fcgamma receptor (FCGR)-dependent phagocytosis (*P* = 0.025; FDR = 0.100) was overrepresented in downregulted DEPs. Regarding C2 treatment, within upregulated DEPs, there were enriched pathways related to heterophil degranulation (*P* = 0.001; FDR = 0.022) and TLRs cascades (*P* = 0.001; FDR = 0.023) at DOH. By 10 days of age, Rap1 pathway (*P* = 0.003; FDR = 0.019) was significant. Within the underexpressed DEPs profile, the FCGR-dependent phagocytosis was enriched at DOH (*P* = 0.0002; FDR = 0.007). By 10 days of age, antimicrobial peptides (*P* = 0.031; FDR = 0.130) and heterophil degranulation (*P* = 0.022; FDR= 0.130) were enhanced.

### Identification of Immune-Related DEPs

Proteins related to immunity among the treatments were ranked based on a functional comparison involving the GO terms. The key immune-related DEPs included leukocyte cell-derived chemotaxin-2 (LECT2), avidin (AVD), high mobility group protein B1 (HMGB1), activated leukocyte cell adhesion molecule (ALCAM), cathelicidin-2 (CAMP), and lysozyme C (LYZ). The DEPs associated with immune function were obtained from the following categories: immune system process, immune response, response to stress, cellular response to stimulus, and related functions (Supplementary Data).

**Figure 6:**
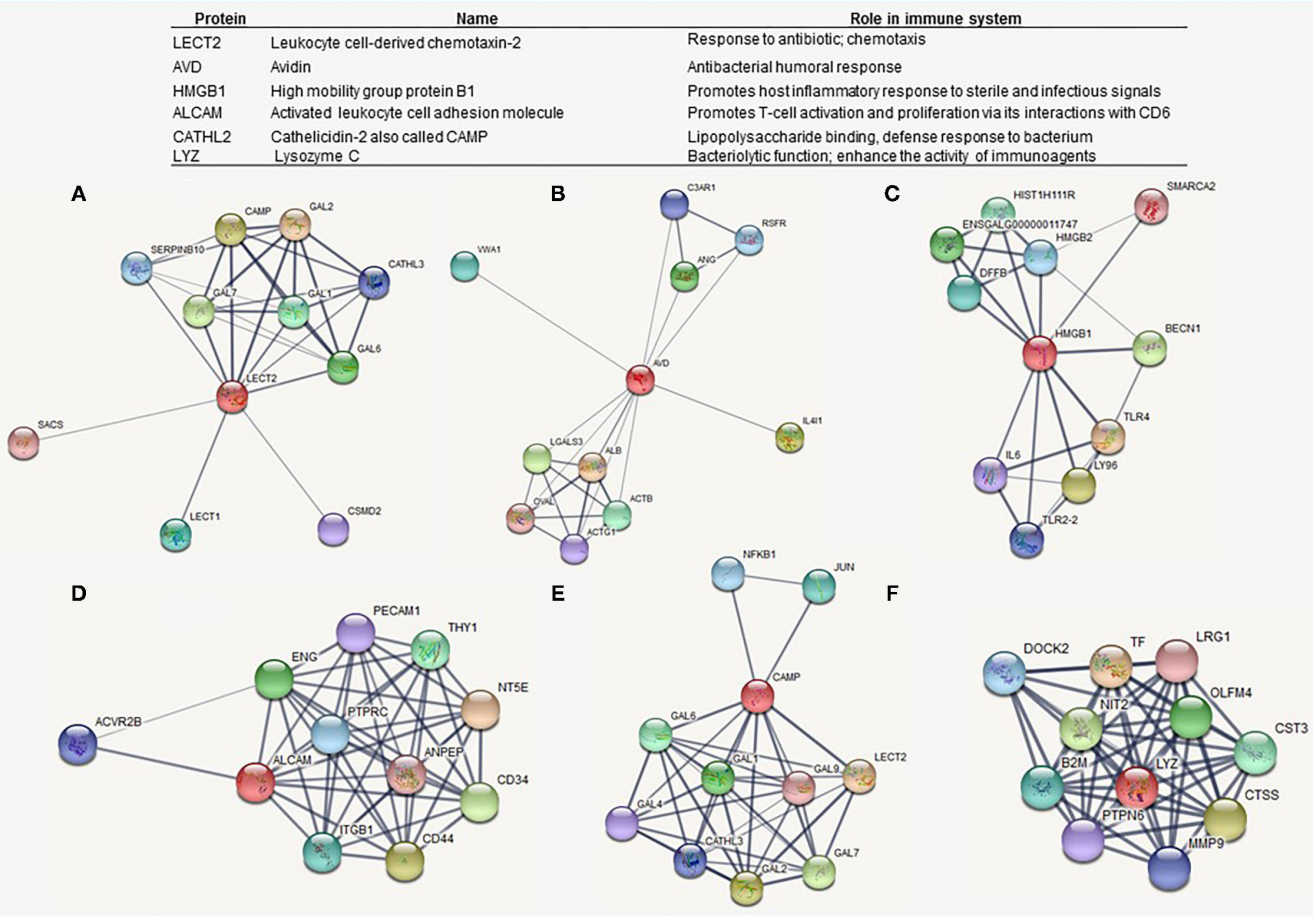
Protein-protein interaction (PPI) network analyses of selected differentially expressed proteins associated with immunomodulatory functions. Red nodes denote the selected immune-related protein, and edges show the biological relationship between nodes. **(A)** Leukocyte cell-derived chemotaxin-2 (LECT2). **(B)** Avidin (AVD). **(C)** High mobility group protein B1 (HMGB1). **(D)** Activated leukocyte cell adhesion molecule (ALCAM). **(E)** Cathelicidin-2 also (CAMP). **(F)** Lysozyme C (LYZ).

Then, a PPI network for each key protein was screened for a better understanding of their roles and underlying mechanisms involving immune processes. Figure 6 shows the interactome networks for LECT2, AVD, HMGB1, ALCAM, CAMP, and LYZ, in which the nodes represent proteins, while the edges are physical, biochemical, or functional interactions between them.

## Discussion

The presence of distinct intestinal proteome profiles in chicks inoculated *in ovo* with *Enterobacteriaceae* isolates or L-probiotic poses the question of whether the expression of intestinal immune-related proteins is dependent on specific early microbiota exposure. Here, we conducted different proteomic approaches as pathway enrichment and GO function annotation analyses to understand the impact of different early life intestinal colonizers on innate and adaptive immune processes in chicks. It was also the focus of this research to identify candidate protein markers for monitoring immune biological functions in broilers. The results of this study provide a significant comprehension of how early exposure to beneficial bacteria may affect immune programming in broilers.

Our previous work also addressed the *in ovo* technique as an experimental model to study how early intestinal colonization can shape the development and persistence of microbiome in chicks (Rodrigues et al., 2020a; Wilson et al., 2020). In those studies, it was shown that different bacterial isolates provided *in ovo* resulted in distinct microbiome profiles on DOH and by 10 days of age. Notably, inoculation of L resulted in increased Lactobacilli populations at DOH, which may have influenced the establishment of butyrate-producing bacteria and segmented filamentous bacterium (SFB) in young broilers. The poultry GIT microbiota undergoes a period of heavy changes during the first days of life. Some reports have shown that the microbiota composition of post-hatched chicks is primarily Proteobacteria derived from opportunistic environmental communities (Ballou et al., 2016; Donaldson et al., 2017; Rodrigues et al., 2020a; Wilson et al., 2020). Concomitant with this process occurs the development, expansion, and education of the mucosal immune system (Chow et al., 2010; Gensollen et al., 2016; Zheng et al., 2020). The adaptive immune functions of newly hatched chicks develop only toward the end of the first week post-hatch (Barshira and Friedman, 2006). Therefore, maternal antibodies and the innate immune system are the main apparatus for dealing with any early pathogenic assault.

From that perspective, early exposures are significant determinants for programming innate immune functions. We found that L treatment enriched the key pathways of the innate immune signaling as heterophil degranulation and antimicrobial peptides at DOH (Figure 5). One of the mechanisms displayed by the innate immune system of the host in reaction to microbial stimulation or into the pathogen-containing phagosome is heterophil degranulation, which releases granule substances including antimicrobial peptides into the external environment (Kogut et al., 2001; Genovese et al., 2013). In agreement with our study, Farnell and Donoghue (2006) reported that oral administration of probiotics could stimulate heterophil oxidative bursting and degranulation in the poultry intestine. Likewise, the upregulated DEPs were predominantly associated with cellular response to stress and external stimuli pathways. Part of cellular responses to external stimuli is carried out by macroautophagy that is considered a cytoprotective host defense mechanism against damaged organelles, cytosolic proteins, and invasive microbes (Feng et al., 2014; Delorme-Axford and Klionsky, 2018). Macroautophagy assists both innate and adaptive immunity releasing lysosomes for the degradation of foreign substances, including pathogenic proteins within cells (Gannagé and Münz, 2009; Bel and Hooper, 2018). Following this process, the products of lysosomal degradation are presented for MHC class II molecules and recognized by CD4+ T cells (Schmid et al., 2007). Correspondingly, there was an enrichment of the MHC class II antigen presentation pathway in L by 10 days of age.

In contrast, these results revealed that the introduction of CF *in ovo* downregulated the MHC class II antigen presentation pathway. In addition, the downregulated DEPs in CF treatment were allied to transport, localization, and response to the stimulus at an early and later age, indicating that the functions associated with the trafficking of immune cells, detection, and response to a biotic and abiotic stimulus may have been impaired. Nedjic et al. (2008) have shown that a change in the MHC class II system by a genetic interference of autophagy in the thymus of mice resulted in severe colitis and multi-organ inflammation. Comparably, our recent research has indicated that pre-hatch *Enterobacteriaceae* colonization perturbed the initial microbial establishment and promoted intestinal proteomic changes accompanied by inflammation in chicks (Wilson et al., 2020). The failure of immune regulation suggested by these results might be connected with the onset of intestinal chronic inflammation signaling in CF treated broilers at 10 days of age (Rodrigues et al., 2020a,b).

Interestingly, we found that C2 overrepresented the regulation of TLRs by endogenous ligand and TLRs receptors cascade pathways at DOH. As feedback mechanisms during either infection or injury to the organism, pathogen-associated molecular patterns (PAMPs) and damage-associated molecular patterns (DAMPs) indicate a danger alert that activates TLRs (Piccinini and Midwood, 2010). Additionally, there was evidence of other innate mechanism activation by the enrichment of the heterophil degranulation pathway in C2 on DOH (Figure 5). Contrarily, the inoculation of C2 *in ovo* has been shown to downregulate the pathway FCGR-dependent phagocytosis at the same age. Phagocytosis is a significant event involving the recognition of invading foreign particles by specific types of phagocytic receptors and the subsequent internalization of the particles (Alberts et al., 2002; Henneke and Golenbock, 2004; Feng et al., 2014). These early events that are mediated by the innate immune system are critical to eliminating the invading infectious agents (Henneke and Golenbock, 2004). The clustering of FCGRs by IgG antibodies on the phagocyte initiates a variety of signals, which lead, through the reorganization of the actin cytoskeleton and membrane remodeling, to the formation of pseudopods and phagosomes (Joshi et al., 2006). As a result of this process, pathogen-derived molecules can be presented on the surface of the host cell, allowing the induction of pathogen-specific adaptive immunity (Alberts et al., 2002; Kumar et al., 2013). Collectively, the underexpression of DEPs on GO terms related to immune defense and the downregulation of pathways related to phagocytosis suggested that C2-treated chicks may have been able to activate the recognition of potentially harmful microorganisms, despite that their defense mechanisms may have failed in executing bacterial clearance. Although we found links between proteomic signatures and the immune system, this study did not assess the link with the host phenotypes. Future research is warranted to evaluate whether immune response-associated proteomic signature can affect body weight and gut health parameters.

We took our results one step further to identify DEPs directly relevant to biological functions associated with early-age immune response in poultry. The mass spec-based proteomics outcomes have been extensively used to discover potential biomarkers to predict or confirm health disorders in human medicine (Geyer et al., 2017). Despite the lack of information, the expansion of modern technologies is beginning to be approached for this purpose in poultry production (Arsenault et al., 2014; Kuttappan et al., 2017). Here, in this study, we suggest a panel of proteins for evaluating mucosal immune response in broilers (Figure 6).

Among the highlighted proteins, LECT2 plays an important role in the immune processes by increasing cytokine expression, inducing chemotaxis, and activating macrophages (Liu et al., 2014; Slowik and Apte, 2017; Jung et al., 2018). Previous work revealed a decreased expression of LECT2 on the proteome of heterophils from the spleen of the chicken in response to *Salmonella* Enteritidis infection. Birds vaccinated against *Salmonella* sp. succeeding a *Salmonella* challenge upregulated granular proteins as CATHL2, also called CAMP, and LECT2, suggesting that these peptides might be considered a positive marker of enhanced immune response to vaccination (Sekelova et al., 2017). Figure 6 demonstrates a co-expression, with a high score (0.976; see Supplementary Data), between LECT2 and CATHL2. Also, LECT2 is strongly related to the GAL1 and other β-defensins, which are a family of peptides with antimicrobial activity and immunomodulatory functions in chickens (Kalenik et al., 2018). In fact, CATHL2 was identified as upregulated in this study along with all treatments, while LECT2 was differentially expressed by L and C2 treatments.

Otherwise, ALCAM and HMGB1 were only upregulated by L treatment, which might have contributed to explaining the activation of immunostimulatory complexes by DOH and 10 days of age, respectively. HMGB1 is actively secreted by innate immune cells in response to PAMPs, where it mediates the activation of innate immune responses, including cytokine release (Yanai et al., 2011; Yang et al., 2013). HMGB1 can also act as a chemotactic mediator by transmitting signals to the cell interior *via* the activation of receptors that include TLR4 (Yang et al., 2013). In relevance of HMGB1 role in cellular and humoral immunity, this protein has been reported as a potential immunological adjuvant in poultry vaccines (Sawant et al., 2015; Yang et al., 2017; Vuong et al., 2020).

Avidin and LYZ are other proteins recently associated with innate antimicrobial activity by preventing direct access of bacteria to the intestinal epithelial surface in newly hatched chicks (Shira and Friedman, 2018). LYZ was found upregulated in L treatment at DOH. Given the potent function of LYZ in limiting the bacterial growth at mucosal surfaces, we speculate that early exposure to L might be a strategy to enhance the innate antimicrobial activity. Finally, further studies are recommended to validate this panel of proteins as potential biological markers of the enhanced immune response in broilers.

Our study indicated that there were predicted biological functions of L-treated chicks associated with the activation and balanced function of the innate and adaptive immune systems. At the same time, the *Enterobacteriaceae* exposed birds presented dysregulated immunological mechanisms or downregulated processes related to immune development. It is expected that the activated response at an early age may manifest the ability of the immune system to recognize, uptake, and destroy foreign microorganisms. Those findings highlighted that a proper immune function was dependent on specific GIT microbiota exposure, in which the prenatal probiotic application may have favored the fitting programming of immune functions in chicks.

## Data Availability Statement

The mass spectrometry proteomics data has been deposited to the ProteomeXchange Consortium *via* the PRIDE partner repository with the dataset identifier PXD015504.

## Ethics Statement

The animal study was reviewed and approved by the Institutional Animal Care and Use Committee (IACUC).

## Author Contributions

KW and DR carried out the project. DR performed the analyses, interpreted the results, and wrote the manuscript in consultation with LB. All authors contributed to the experimental design, discussed the results, and commented on the manuscript.

## Funding

This research was supported by the OARDC Research Enhancement Competitive Grants Program (SEEDS) (Grant No. 2016035) and Arkansas Biosciences Institute (ABI: Little Rock, AR).

## Conflict of Interest

The authors declare that the research was conducted in the absence of any commercial or financial relationships that could be construed as a potential conflict of interest.

## Publisher's Note

All claims expressed in this article are solely those of the authors and do not necessarily represent those of their affiliated organizations, or those of the publisher, the editors and the reviewers. Any product that may be evaluated in this article, or claim that may be made by its manufacturer, is not guaranteed or endorsed by the publisher.
